# An elastic segment of the whisker shaft enables coding of the whisking phase via whisker torsion in rats and mice

**DOI:** 10.1002/ar.70051

**Published:** 2025-09-09

**Authors:** Sebastian Haidarliu, Guy Nelinger, Luka Gantar, Ehud Ahissar, Inbar Saraf‐Sinik

**Affiliations:** ^1^ Department of Brain Sciences The Weizmann Institute of Science Rehovot Israel; ^2^ Neuroethology Research Group Institute of Science and Technology Klosterneuburg Austria

**Keywords:** elastic whisker segment, encoding whisking phase, mechanoreceptors, reafference

## Abstract

Rodents' ability to encode the whisking phase has been extensively documented through neuronal recordings from ascending sensory pathways. Yet, while indicating that reafference originates from the mechanoreceptors, the mechanistic underpinnings of the whisking phase encoding within the follicle remain unclear. Here we present anatomical, histological, and biomechanical evidence for the presence of a distinctive elastic segment (ES) within the basal part of the whisker shaft inside the follicle. This ES, composed of immature keratin, is capable of both bending and twisting. Forces generated by whisker movement deform this segment, causing whisker shaft deflections that can stimulate specific mechanoreceptor subsets within the follicle at different phases of the whisking cycle. This mechanism appears to operate during both free‐air whisking and object contact. We propose that the ES enables torsion‐based mechanoreceptor activation, allowing encoding of the whisking phase.

## Graphical abstract

An elastic segment was found in the basal part of the whisker shaft in rats and mice. Application of force to the whisker bulb of isolated follicles caused bending and twisting of this segment. Active whisker movements deform this segment, causing whisker shaft deflection and selective activation of mechanoreceptors at different phases of whisking.

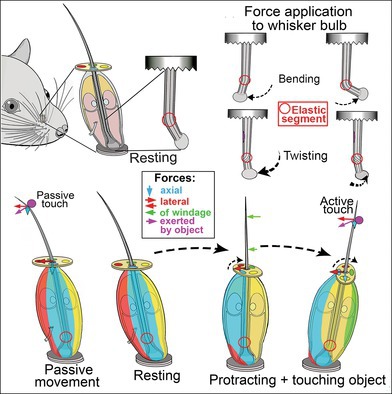

**Abbreviations**
ES: elastic segment
MP: mystacial pad
SFM: subcapsular fibrous mat

## 1 INTRODUCTION

Nocturnal, burrowing rodents rely heavily on their whiskers to navigate, identify nearby objects, hunt, and interact socially (Grant et al., 2018; Sofroniew & Svoboda, 2015). Using rhythmic whisking movements, they determine the azimuthal position of objects by integrating information about contact events and the whisking phase at the moment of touch.

While extensive evidence confirms that neurons in the trigeminal ganglion (Campagner et al., 2016; Gibson & Welker, 1983; Khatri et al., 2009; Leiser & Moxon, 2007; Lichtenstein et al., 1990; Severson et al., 2017, 2019; Shoykhet et al., 2000; Szwed et al., 2003; Zucker & Welker, 1969), brainstem (Ebert et al., 2021; Furuta et al., 2008; Mohar et al., 2013; Moore et al., 2015; Wallach et al., 2016; Xiang et al., 2014), thalamus (Brown & Waite, 1974; Moore et al., 2015; Oram et al., 2024; Sumser et al., 2025; Urbain et al., 2015; Yu et al., 2006, 2015), and barrel cortex (Andermann & Moore, 2006; Castro‐Alamancos & Bezdudnaya, 2015; Curtis & Kleinfeld, 2009; de Kock & Sakmann, 2009; Fee et al., 1997; Ganguly & Kleinfeld, 2004; Isett & Feldman, 2020; Jadhav & Feldman, 2010; Kremer et al., 2011; Kwon et al., 2018; Simons, 1978; Tsytsarev et al., 2010; Vilarchao et al., 2018) encode whisking phase, the biomechanical basis of this coding at the level of the follicle remains poorly understood. Specifically, how physical forces during whisking selectively activate mechanoreceptors at different phases of the cycle is still unresolved.

Previous anatomical and mechanical studies have described the follicle as a mostly rigid structure due to its collagen capsule. The capsule maintains a high internal hydrostatic pressure, which is believed to prevent mechanical deformation (Melaragno & Montagna, 1953; Rice et al., 1986; Scott, 1955; Stenn et al., 1988). However, some evidence points to localized softness at the lower level of the cavernous sinus (Ebara et al., 2002), a thinner capsule that surrounds its proximal end (Kim et al., 2011), and the flexibility of the internal part of the whisker shaft (Whiteley et al., 2015). These conflicting findings highlight the need for further insight into the follicular mechanical properties and their contribution to sensory coding.

Here, we distinguish two functionally different parts of the whisker: the *external* part, which interacts with the environment, and the *internal* part, which resides within the follicle and relays forces received by the external part to the mechanoreceptors. The internal part consists of a distal rigid portion (approximately two thirds) that contains mature keratin and a proximal intermediate portion adjacent to the whisker bulb where keratin synthesis and maturation occur (Lee & Coulombe, 2009; Rogers, 2004).

Although previous analyses have focused mainly on the geometry, mechanical properties, and kinematics of the external part of the whisker shaft in three‐dimensional space (Belli et al., 2017, 2018; Knutsen et al., 2008; Quist et al., 2011; Towal et al., 2011), it is the internal part of the whisker shaft that plays a direct role in encoding the whisking phase. Based on known histological features and keratin maturation dynamics (Ibrahim & Wright, 1975; Larouche et al., 2008), we predicted the existence of a flexible segment within the basal part of the intermediate portion of the whisker shaft. Through anatomical dissection, mechanical manipulation, and histological staining, we found a flexible segment that undergoes torsional and bending deformation during force application to the whisker bulb. Since the whiskers perform both angular and torsional movement, this deformation will drive the selective stimulation of subsets of mechanoreceptors at different phases of the whisking cycle. While our experiments were conducted ex vivo, our findings suggest a biomechanical mechanism for phase encoding that bridges follicular anatomy with whisker dynamics.

## 2 MATERIALS AND METHODS

### 2.1 Animals

Eight adult male Wistar rats and seven adult mice, three from the Hsd:ICR and four from C57BL/6 lines, were used in the experiments. Skull and mystacial pad (MP) samples for microdissection and histology were collected from rats and mice sacrificed for experiments unrelated to the present report, which were performed in accordance with the guidelines of the Institutional Animal Care and Use Committee at the Weizmann Institute of Science.

### 2.2 Video recordings

Video recordings of freely behaving rats were performed under infrared (IR)‐only illumination (880 nm) using a high spatiotemporal resolution camera (Optronis, 1024 × 1280 pixels, 500 Hz). The camera was attached to the top of the permanent living space. Behavioral dynamics were recorded and analyzed.

### 2.3 Anatomy and histology

The mechanical properties of the whisker shaft were studied after their partial exposure in follicles separated from the MP. In the MP, the follicles are attached to muscles and connective tissue, which are soft and stringy, making it difficult to separate the follicles. Since tissue fixation reduces tissue elasticity (Ling et al., 2016), we used only dehydration with 15% sucrose in phosphate buffered saline (PBS, pH 7.4), which resulted in the MP sinking to the bottom in approximately 24 h at 4°C. This moderate dehydration did not cause significant tissue shrinkage, but it did lead to muscle hardening and facilitated muscle removal, while maintaining the MP follicles in a “life‐like” state. The capsule was mechanically cleaned and cut only at the very tip of the whisker bulb due to its adhesion to the subcapsular fibrous mat (SFM).

**FIGURE 1:**
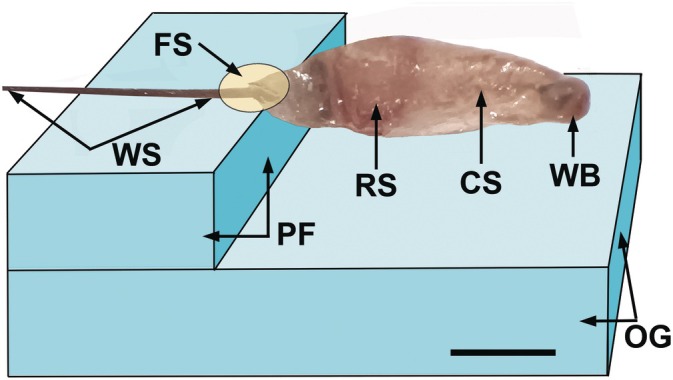
Device for horizontal fixation of follicles. CS, cavernous sinus; FS, fixation site; OG, object glass; PF, plate for follicles' support; RS, ring sinus; WB, whisker bulb; WS, whisker shaft. Scale bar = 1 mm.

Whisker shaft exposure was performed in two stages using a microscope, a needle, and a microsurgical knife (Pearsalls Limited, UK). First, the capsule, blood, and trabeculae of the cavernous sinus were removed from about a third of the isolated follicles, starting from their proximal ends and exposing the whisker bulb and whisker shaft surrounded by glassy membrane and two root sheaths. Then, this surrounding tissue was removed, revealing the whisker shaft containing mostly keratin. To hold the follicle in the air in a horizontal plane, the base of the whisker shaft and the distal end of the follicle were fixed with the ventral surface down using a simple device (Figure 1) and superglue (Loctite Super Glue, Henkel).

Force was applied to the whisker bulb in azimuthal and vertical planes by using a stainless steel wire (125 μm in diameter) or an insulin syringe needle (OMG‐INS‐30G516‐F) attached to the 3D mechanical micromanipulator (Narishige, Japan). The tip of the wire was sharpened to prevent it from slipping off the smooth and bare surface of the whisker bulb. The bending and twisting in the intermediate portion of the whisker shaft during force application were monitored using a binocular microscope with a top light (Labomed Digi Star, Leonhard Instrument Co, Mars, PA, USA), which made it possible to observe when the bending or twisting process was limited to the flexible segment and to stop applying force when the entire exposed whisker shaft began to move. While applying force to the whisker bulb in the ventral and dorsal directions, a small spot on the caudal surface of the distal portion of the exposed whisker shaft was first marked with a surgical skin marker (Gentian Violet ink, Aspen Surgical, Caledonia, MI, USA) to observe the torsion of the whisker shaft. The procedures used for staining for cytochrome oxidase activity and revealing autofluorescence have been previously described in detail (Haidarliu et al., 2011).

## 3 RESULTS

To investigate the mechanical basis of whisking phase encoding within the follicle, we examined the shape and deformation of the internal portion of the whisker shaft. Our anatomical and mechanical observations identified a distinct segment within the follicle, termed the elastic segment (ES), characterized by high flexibility and immature keratin content. In isolated follicles, forces mimicking free‐air whisking caused localized bending and twisting of the ES, displacing the shaft and altering internal deflection patterns. These deformations may differentially engage the circumferentially organized follicular mechanoreceptors. The following sections detail the ES's identification, mechanical properties, and proposed role in whisking phase encoding.

### 3.1 Detection of the elastic segment of the whisker shaft

**FIGURE 2:**
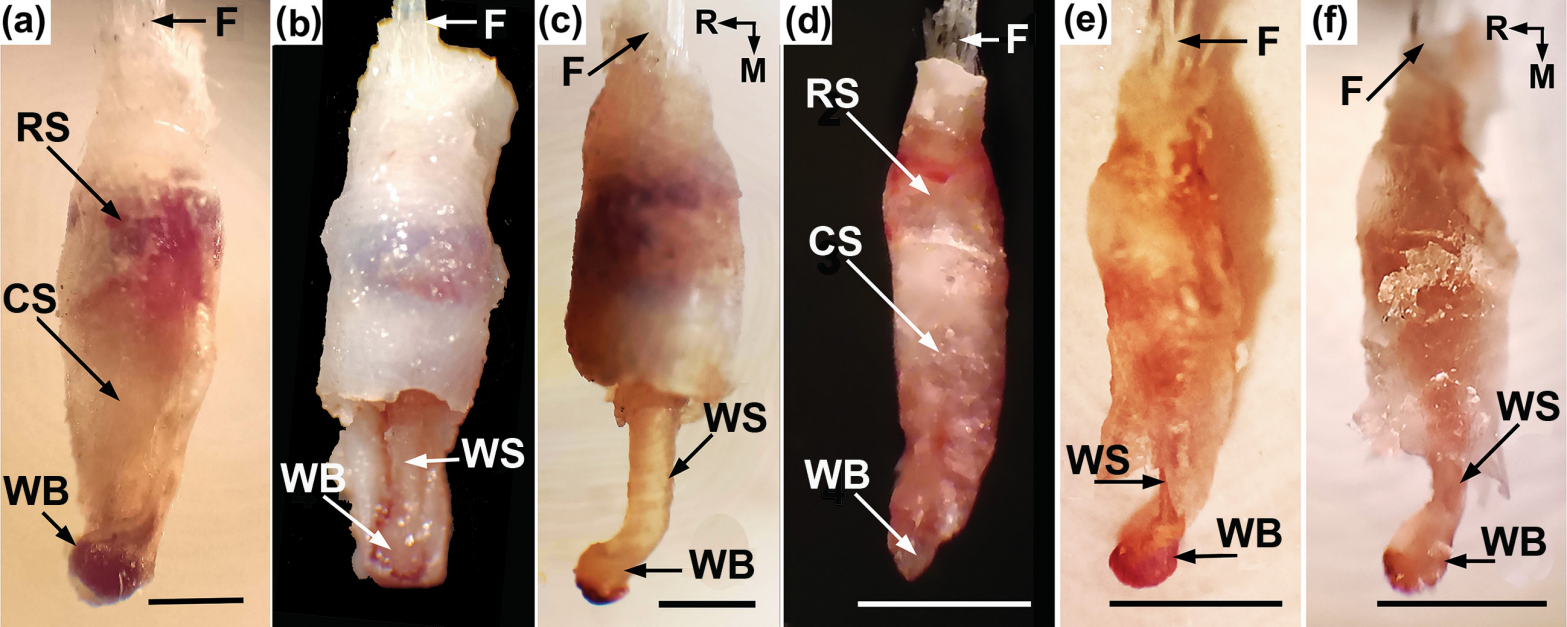
Inherent bend of the proximal ends of large follicles and whisker shafts in rats (a–c) and mice (d–f). (a and d) Isolated follicles; (b and e) follicles with partially removed capsule; (c and f) follicles with exposed whisker bulb and a part of the whisker shaft. CS, cavernous sinus; F, fixation site; M, medial; R, rostral; RS, ring sinus; WB, whisker bulb; WS, whisker shaft, composed of keratin and covered by two root sheaths and a glassy membrane. Scale bars = 0.5 mm.

The whisker shaft acquires environmental stimuli when mechanical forces act on its external part and are transmitted to the internal part. This transmission creates shear forces at the corium level and output forces at the proximal end represented by the whisker bulb. These output forces induce tension in the internal part of the whisker shaft, causing it to deform and thereby stimulate mechanoreceptors. However, this deformation is normally obscured by surrounding tissues, including the collagen capsule and blood filling the sinuses. To reveal the location of such deformation, we partially exposed the internal whisker shaft by removing the capsule, blood, and trabeculae of the cavernous sinus from the proximal third of isolated large follicles that consistently exhibited a rostrally directed bend at their proximal ends, mirrored by the exposed whisker shafts (Figure 2).

**FIGURE 3:**
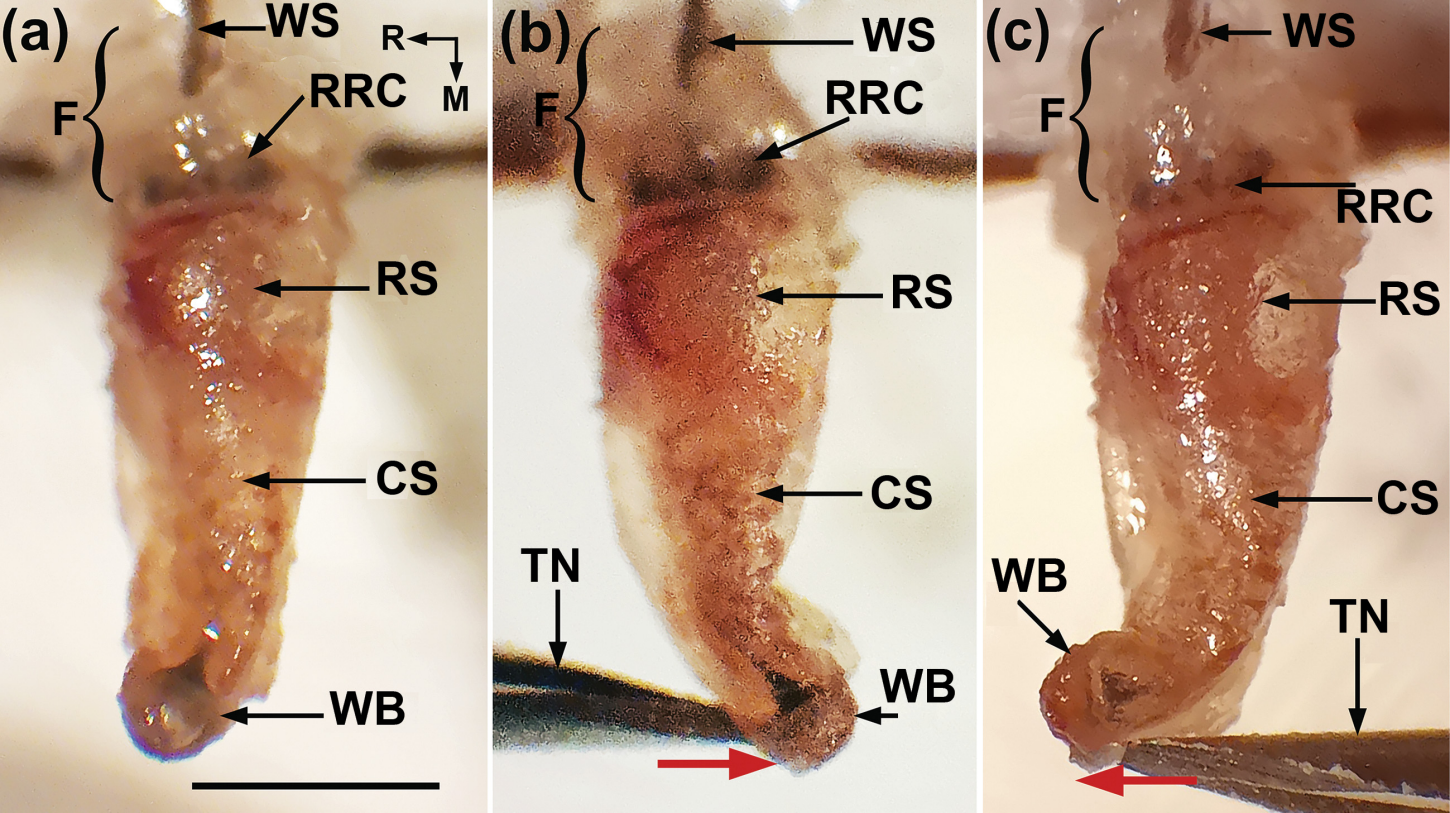
Force application to the whisker bulb of the isolated follicle D2 in the MP of the C57BL/6 mouse. (a) Isolated follicle in steady position; (b and c) force application in caudal and rostral direction, respectively. CS, cavernous sinus; F, fixation site; M, medial; R, rostral; RRC, rete ridge collar; RS, ring sinus; TN, tip of the insulin syringe needle; WB, whisker bulb; WS, whisker shaft. Red arrows indicate directions of force application. Scale bar = 0.5 mm.

**FIGURE 4:**
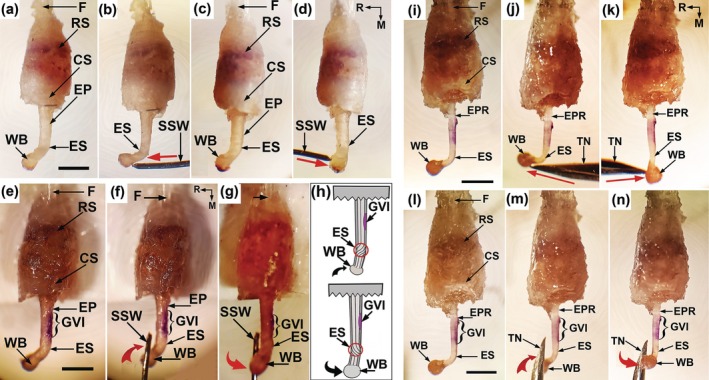
Force application to the whisker bulb of exposed whisker shafts of the follicle C2 (a–d) and D2 (e–n) in rats. (a, c, e, i, and l) Whisker shafts in steady position; (b and j) force application in rostral direction; (d and k) force application in caudal direction; (h) cartoon showing the twisting of the ES in (f) (top) and (g) (bottom). CS, cavernous sinus; EP, exposed part of the whisker shaft covered by two root sheaths and glassy membrane; EPR, exposed part of the whisker shaft in which the two root sheaths and glassy membrane were removed; ES, elastic segment; F, fixation site; GVI, Gentian violet ink; M, medial; R, rostral; RS, ring sinus; SSW, stainless steel wire; TN, tip of the insulin syringe needle; WB, whisker bulb. Red arrows indicate directions of force application. Scale bars = 0.5 mm.

We applied directional forces to the whisker bulb of these isolated follicles under three conditions: with the capsule preserved intact (Figure 3), after removing the capsule, blood, and trabeculae from the proximal third of the follicle (Figure 4a–g), and after removing the glassy membrane and both root sheaths, leaving only the keratin‐based shaft (Figure 4i–n). In all cases, the follicle distal end and the whisker base were fixed in place (see Figure 1).

Forces applied to the whisker bulb revealed a localized bending zone in the basal part of the intermediate portion of the whisker shaft (Figure 4). Specifically, force in the rostral direction altered the whisker's curvature, reducing its obtuseness (Figure 4b,j), and force in a caudal direction straightened the whisker shaft (Figure 4d,k). Importantly, this bending was consistently confined to a distinct region, even though the entire internal shaft was exposed to buckling forces, indicating a localized increase in compliance. We refer to this deformable region as the ES.

During natural whisking, the whiskers move in the azimuthal plane, simultaneously undergoing axial rotation (torsion). This torsion is expected to cause the bending plane of the ES to shift—from azimuthal at the retraction set point to approximately vertical at the protraction set point. To examine how the ES responds to windage forces during protraction, accompanied by whisker torsion, we simulated this condition by applying force to the whisker bulb in the dorsal and ventral directions (Figure 4f,g). These manipulations resulted in twisting of the shaft in the same area that we designated as ES. A spot marked with Gentian Violet ink remained in the same position regardless of whisker deformation, confirming that twisting was confined to the ES. Notably, this deformation was reversible, as upon force release, the whisker shaft quickly returned to its original shape, suggesting the ES endows elasticity to the shaft.

As can be seen in Figure 4, the deformation of the whisker shaft upon application of force was approximately the same both when it was covered by two root sheaths and a glassy membrane and when all these sheaths were removed. This suggests that the local flexibility of the whisker shaft is mainly due to the presence of immature keratin within the ES.

### 3.2 Immature keratin of the ES endows its elasticity and facilitates changes of the whisker shaft shape

**FIGURE 5:**
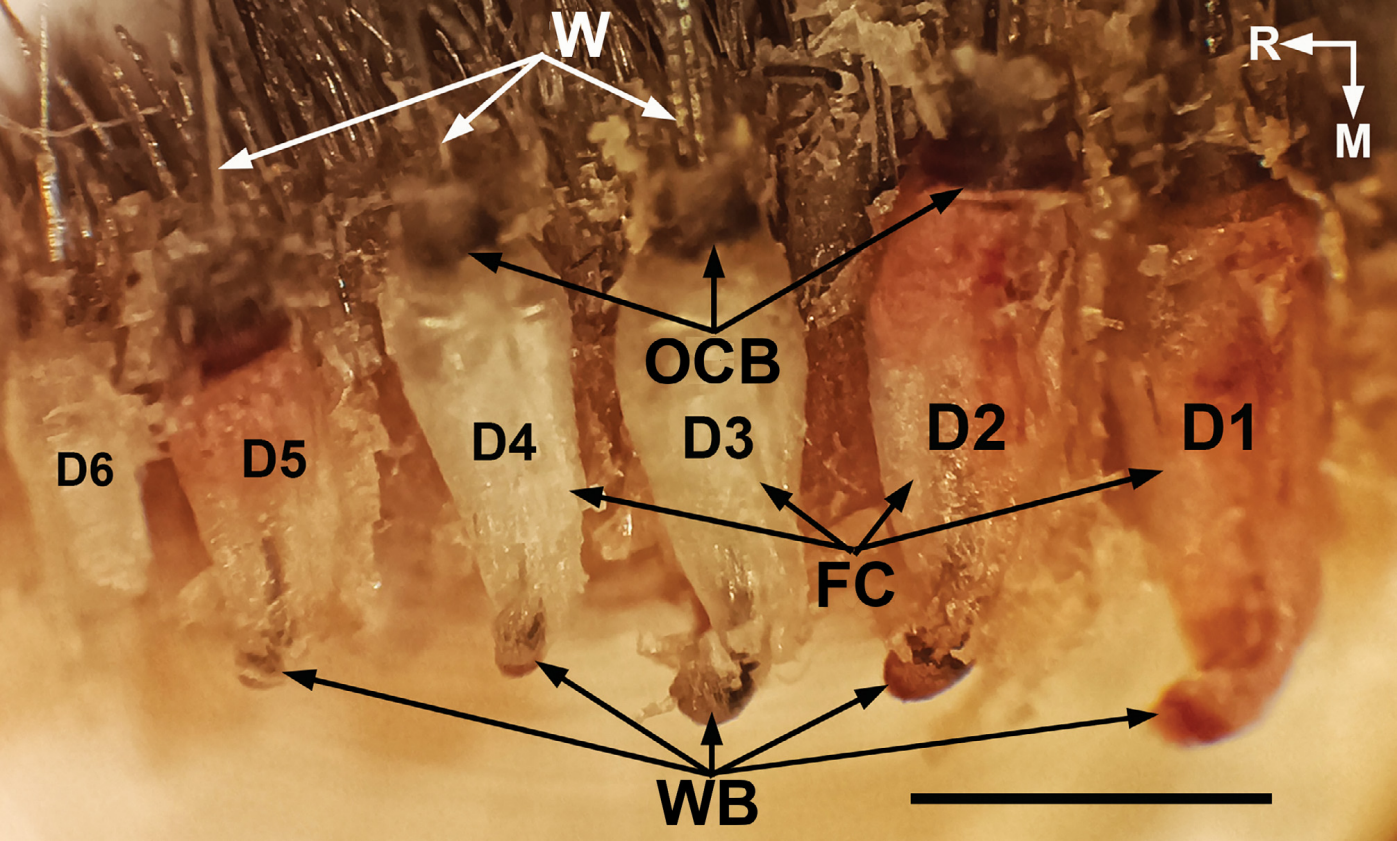
Exposed follicles in the MP of a C57BL/6 mouse. D1 to D6, follicles of the row D; FC, follicular capsules; M, medial; OCB, outer conical bodies; R, rostral; W, whiskers; WB, whisker bulbs. Scale bar = 1 mm.

**FIGURE 6:**
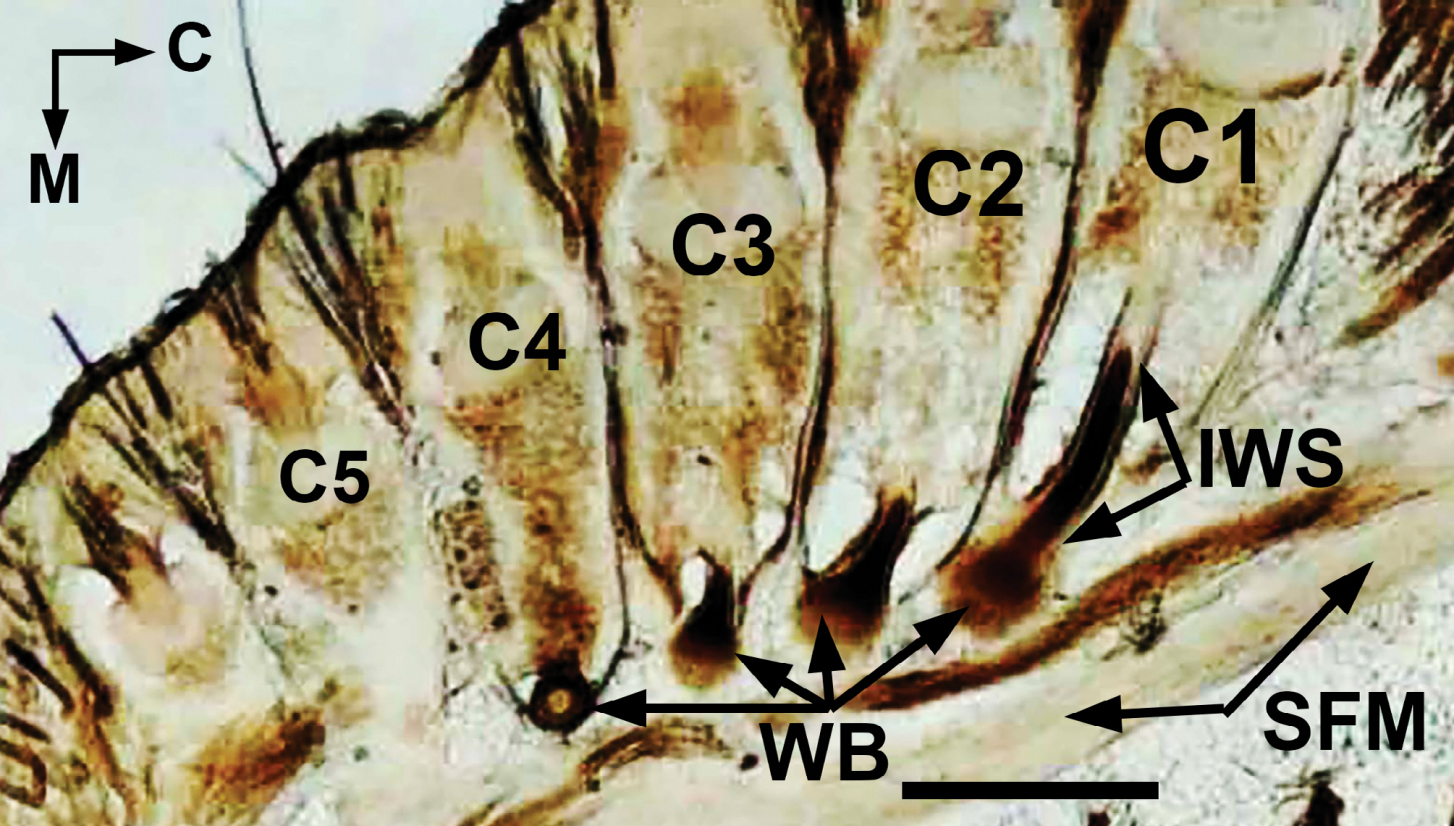
A horizontal slice (60 μm thick) of an adult rat MP stained for cytochrome oxidase activity. C, caudal; C1 to C5, follicles of the row C; IWS, intermediate portion of the whisker shaft; M, medial; SFM, subcapsular fibrous mat; WB, whisker bulbs. Scale bar = 1 mm.

Anatomically, whisker follicles exhibit systematic morphological variation across the MP. Macrovibrissae, located in the caudal part of the MP, gradually decrease in size along the rows in the rostral direction (Brecht et al., 1997). We found that the degree of the inherent follicle's curvature also varies across different regions of the MP. In mouse row D (Figure 5) and rat row C (Figure 6), the proximal ends of follicles displayed a graded rostrally directed curvature, which was more pronounced in the caudal whiskers. This curvature gradient aligned with the location of the ES, raising the possibility that local shaft curvature modulates the mechanical behavior of the ES across whiskers.

**FIGURE 7:**
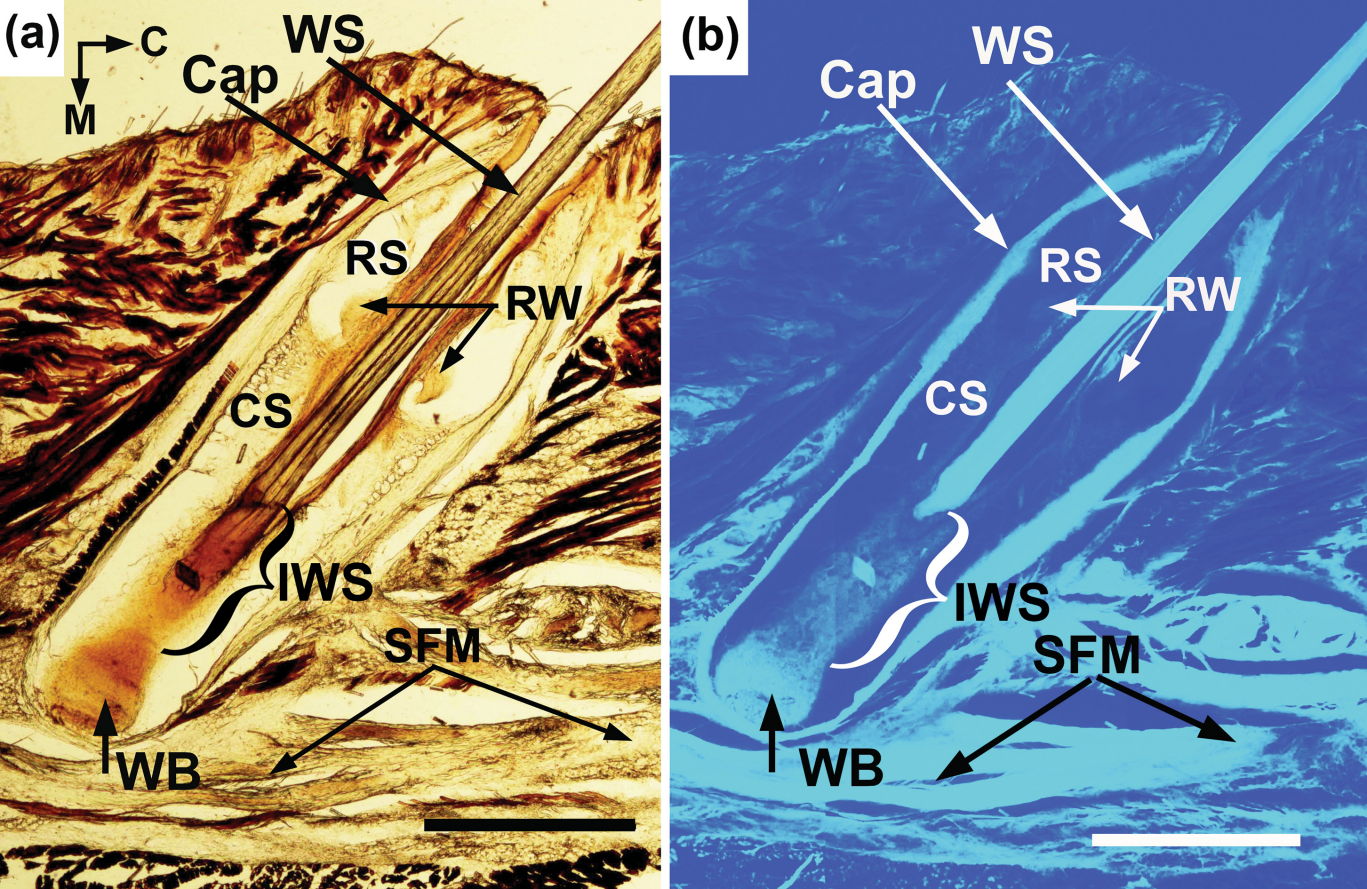
A lengthwise slice (60 μm thick) of the follicle C4 in the MP of an adult rat. (a) Staining for cytochrome oxidase activity; (b) autofluorescence of collagen and keratin in the same slice. C, caudal; Cap, capsule; CS, cavernous sinus; IWS, intermediate portion of the whisker shaft; M, medial; RS, ring sinus; RW, ringwulst; SFM, subcapsular fibrous mat; WB, whisker bulb; WS, whisker shaft. Scale bar = 1 mm.

To better understand the structural basis for this localized flexibility, we examined the molecular composition of the ES. Histological staining and autofluorescence imaging confirmed that the ES is compositionally distinct from adjacent shaft regions. Cytochrome oxidase staining revealed stronger staining in the intermediate portion of the shaft (Figure 7a), consistent with a higher content of immature keratin, compared to the distal part, which contains mature keratin.

Autofluorescence analysis further distinguished the ES region: while the follicular capsule and SFM that are composed of collagen (Muir et al., 2022), a keratin‐like protein, had a strong autofluorescence, the intermediate shaft exhibited weak autofluorescence (Figure 7b), consistent with its immature keratin and sparse collagen fibers content (Sakita et al., 1994).

### 3.3 The interaction of rigid and elastic anatomical structures of the MP contributes to the encoding of the whisking phase

**FIGURE 8:**
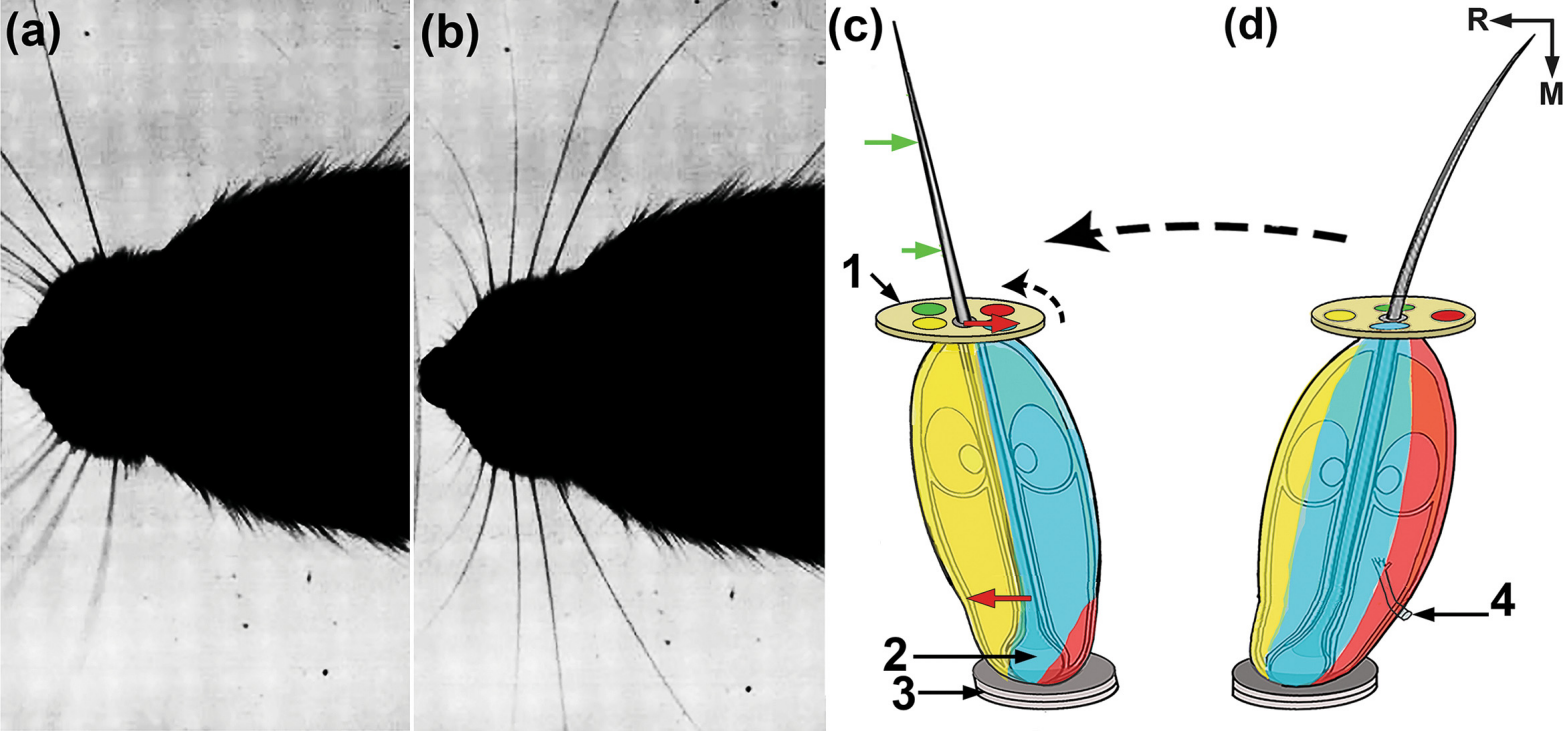
Protraction and torsion of whiskers during free air whisking in a rat. (a and b) Whisker profiles at protraction and retraction set points, respectively. (c and d) Cartoon illustrating the mechanical features that occur in whisker follicles in (a) and (b), respectively (the length of the whisker is not to scale). (1) Corium; (2) whisker bulb; (3) subcapsular fibrous mat; (4) deep vibrissal nerve. M, medial; R, rostral. Green arrows indicate the forces developed by air resistance, red arrows ‐ lateral forces.

Based on these converging anatomical and mechanical observations, we infer that the site of maximal bending corresponds to the ES, located midway along the basal part of the intermediate portion of the whisker shaft. The ES appears to play a key role in translating whisker motion into mechanical signals within the follicle. During natural whisking, whiskers move azimuthally while simultaneously undergoing torsion. High‐resolution video recording of the whiskers' movement during the whisking cycle confirms that whisker curvature changes, reflecting torsional motion. At protraction, most of the large whiskers appear convex in the caudal direction or straight (Figure 8a,c), whereas when retracted, they become concave (Figure 8b,d). As torsion increases during protraction, the ES bends and twists, displacing the distal shaft inside the follicle in a direction that likely shifts the locus of deflection against the follicular wall. These dynamics may sequentially engage radially organized populations of mechanoreceptors.

During whisker protraction, air resistance exerts pressure on the whisker's rostral surface, which generates lateral forces at the level of the whisker bulb. The bulb, held by the SFM, remains stationary, so these forces create tension along the whisker shaft that deforms the ES (Figure 8c). This shifts the distal two thirds of the internal part of the whisker shaft rostrally, stimulating mechanoreceptors of the deep vibrissal nerve. Simultaneously, caudally directed lateral forces at the corium activate mainly the Merkel endings, innervated by the superficial vibrissal nerve. The directions of bending moment and whisker shaft deflection are maintained in the azimuthal plane, while simultaneous torsion rotates the whisker‐follicle assembly, exerting pressure of the deflected whisker shaft sequentially on different sets of mechanoreceptors, encoding the whisking phase. When the muscles relax and the whisker retracts, the elastic energy stored in the ES is released, quickly restoring the shape of the whisker shaft.

**FIGURE 9:**
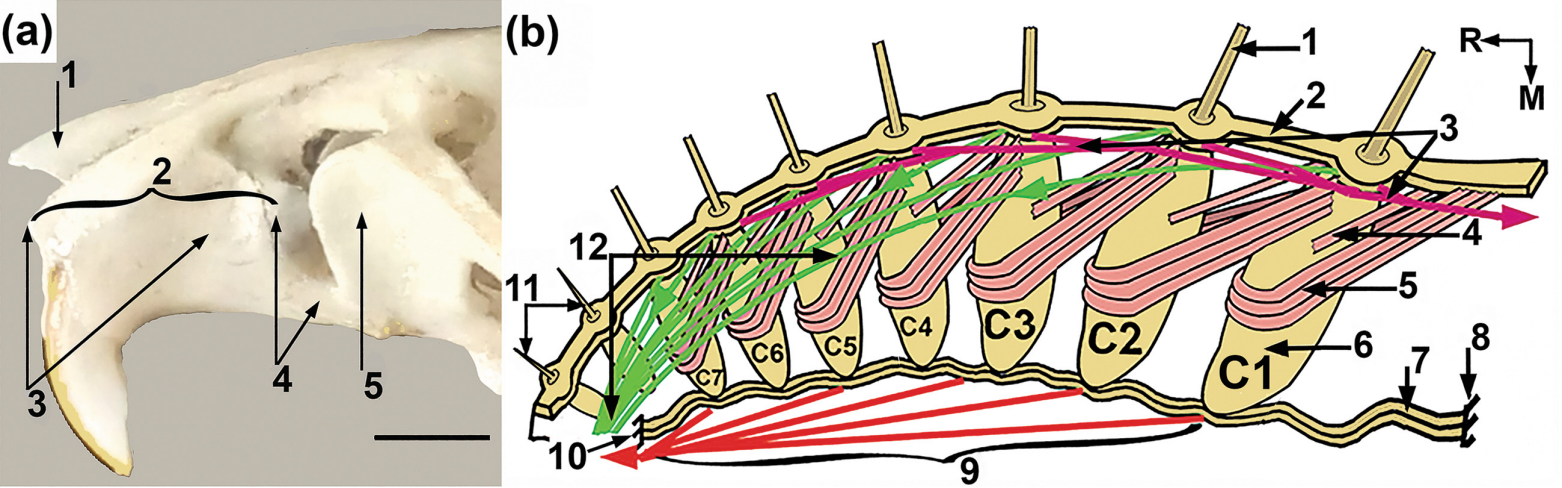
Attachment of the rat mystacial pad (MP) to the skull. (a) Rostral part of the rat skull. (1) Nasal bone; (2) span of the lateral depressed surface (fossa) of the rat skull; (3) premaxilla; (4) maxilla; (5) zygomatic plate. Scale bar = 5 mm. (b) Cartoon showing sites of the rat MP attachment to the skull. (1) Whisker; (2) corium; (3) superficial whisker retractors (nasolabialis and maxillolabialis muscles); (4 and 5) oblique and follicular intrinsic muscles, respectively; (6) follicle; (7) subcapsular fibrous mat (SFM); (8 and 10) caudal and rostral entheses of the SFM, respectively; (9) deep extrinsic whisker retractors (maxillary parts of the nasolabialis profundus muscle); (11) microvibrissae; (12) extrinsic whisker protractor (inferior medial part of the nasolabialis profundus muscle). M, medial; R, rostral.

The structural design of the MP supports this mechanical function. The follicle base is held in place by the SFM, a dense collagenous structure anchored to the skull (Figure 9). This arrangement allows for tight control of whisker positioning while protecting internal structures. Each whisker thus acts as a keratin‐based lever: the external shaft is homogenous, rigid, and composed of mature keratin, while the internal shaft is heterogeneous, integrating a rigid distal portion and a flexible proximal portion containing ES. This biomechanical dynamics position the ES as a potential substrate for encoding the whisking phase—through controlled elastic deformations that modulate the internal shaft geometry relative to the follicular mechanoreceptors.

We assume that the forces arising from the natural protraction of the whisker cause a similar deformation of the internal part of the whisker shaft. Bending and twisting of the ES cause an azimuthal deviation of the rigid distal part of the whisker shaft inside the follicle, activating subgroups of mechanoreceptors whose position corresponds to the degree of torsion and reflects the current phase of the whisking cycle. During whisker retraction, the release of elastic energy stored in the ES, follicular capsule, and its attachment sites to the corium and SFM causes rapid reverse torsion. As a result, the whisker shaft returns to its original shape, maintaining its geometry across cycles.

## 4 DISCUSSION

The whisker follicle–sinus complex is often conceptualized as a mechanical structure that transduces whisker motion into neural signals. Here, we provide a mechanistic explanation for how this transduction occurs, focusing on the deformation of the internal part of the whisker shaft during whisking and object touch and on how these mechanical processes may shape the follicular morphology.

### 4.1 Mechanical basis of signal transduction in the follicle

Active whisking is driven by movements of the follicle, to which the internal part of the whisker shaft is anchored. Upon contact with objects, the external part of the whisker shaft transmits collision‐induced forces inward. The way these forces propagate is determined by the whiskers' material properties, including the geometry of keratin and medullary structures (Belli et al., 2017; Voges et al., 2012). Because the whisker tapers toward its tip, its bending rigidity decreases by over five orders of magnitude from base to tip (Pammer et al., 2013), creating a contact‐specific bending profile shaped more by whisker mechanics than by object properties. The whiskers are characterized by a rostro‐caudal gradient of their base diameter and medulla parameters (Belli et al., 2017), which can influence whisker movements and encoding tactile information due to the mechanical coupling of adjacent follicles through the skin with the possible contribution of the intrinsic muscles connecting follicles (Ego‐Stengel et al., 2019). To understand how mechanical cues lead to the stimulation of specific mechanoreceptors within the follicle, we examined changes in the shape of the internal part of the whisker shaft. It has previously been shown that mechanoreceptors—specifically Merkel cells—shift their position during passive whisker deflection, indicating internal motion (Whiteley et al., 2015). A modeling study predicted that whisker deflection generates an S‐shaped bend of its internal part, crossing the midline of the follicle just below the ring sinus (Luo et al., 2021). However, in this model, the distal part of the whisker shaft showed maximum bending inside the follicle near the corium, where it is rigid (Bagdasarian et al., 2013), while its proximal part remained straight, although it was shown that it could bend (Whiteley et al., 2015).

By applying directional forces to the exposed whisker bulb while the external whisker part was immobilized, we localized this flexibility to a specific internal segment—the ES. Forces applied in the rostral or caudal directions caused the ES to bend, while vertical force resulted in its twisting. Under natural conditions, whiskers experience both angular and torsional motion, creating shear and axial forces (Pammer et al., 2013). As whiskers contact objects, axial forces compress the shaft into the follicle, while lateral forces act along the rete ridge collar, generating output forces at the whisker bulb.

Because axial force and bending moment change inversely during object touch (Pammer et al., 2013, Figure 10A), a closer object position results in a stronger ES bend and a greater deflection of the internal part of the whisker shaft, thereby recruiting more mechanoreceptors. Since this deflection is directionally consistent during whisker torsion, spatially defined mechanoreceptor subsets are sequentially activated in patterns corresponding to the degree of torsion. The change in the shape of the ES when touching an object is accompanied by the accumulation of elastic energy, which is released after the stimulus cessation, returning the whisker shaft to its original configuration.

**FIGURE 10:**
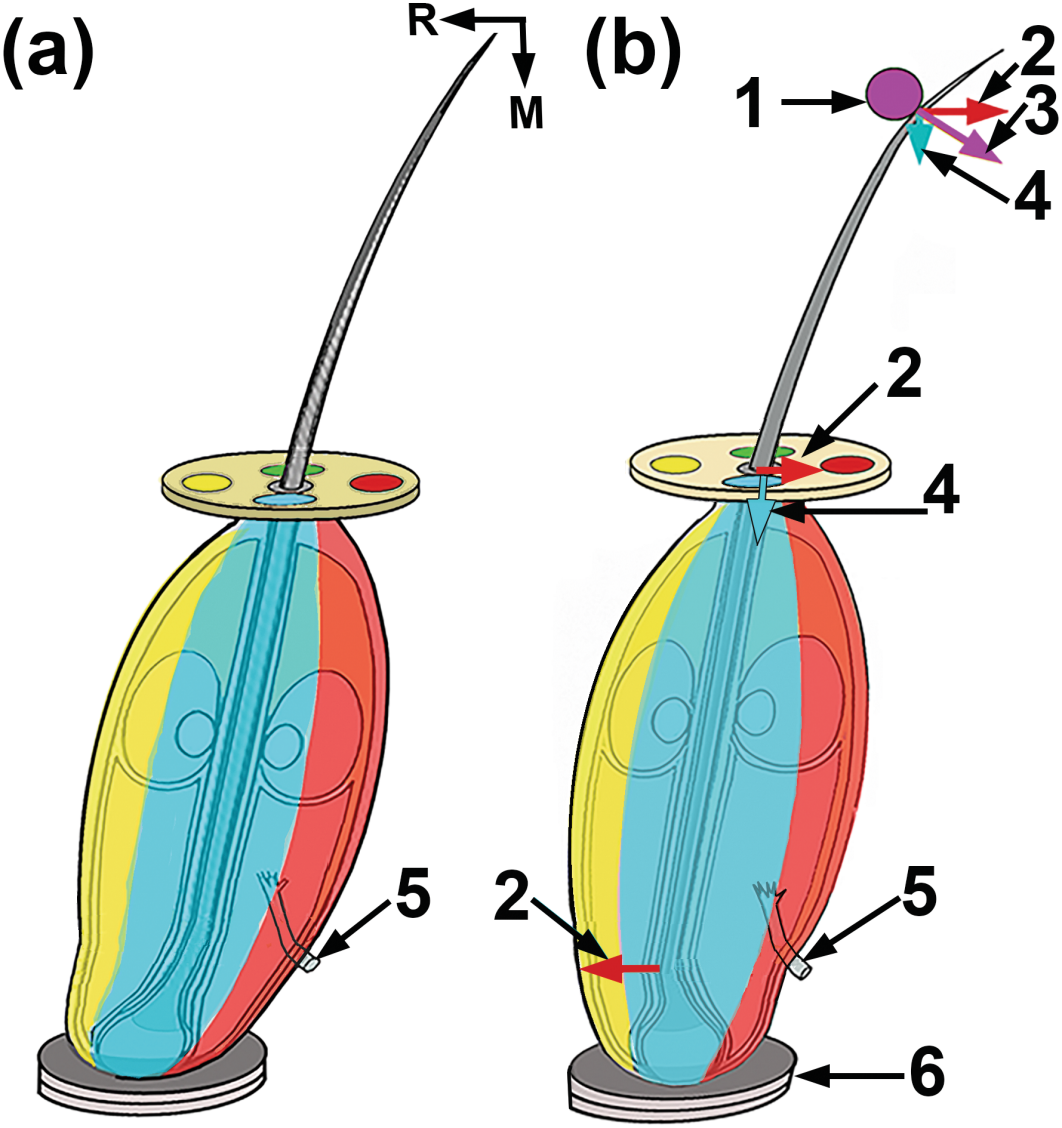
Cartoon illustrating changes in the shape of the whisker shaft during passive movement. (a) Resting whisker; (b) object touch in the azimuthal plane. (1) Object; (2) lateral force; (3) force exerted by the object; (4) axial force; (5) deep vibrissal nerve; (6) subcapsular fibrous mat. M, medial; R, rostral.

Although our experiments were performed on exposed and isolated follicles, we propose that similar force patterns occur under physiological conditions, though likely dampened by the blood‐filled follicle and the viscoelastic properties of the surrounding tissues. Thus, during protraction, the ES bends or twists as a function of azimuthal angle and torsion, steering the rigid distal portion toward the circumferentially arranged mechanoreceptors. Their spatial organization within the follicle enables labeled line whisking phase encoding by the specific mechanoreceptors' subset. The relative flexibility of the follicle's proximal end (Ebara et al., 2002) may also contribute to the rapid recoil of the whisker upon retraction onset, returning the follicle and whisker shaft to their original shape and maintaining their geometry across cycles.

### 4.2 Encoding active versus passive whisker movements

The importance of torsional movement in whisking phase coding becomes particularly clear when comparing active versus passive whisker movements. Active whisking has been studied in freely behaving animals (Grant et al., 2012; Knutsen et al., 2005; Mitchinson et al., 2007; Moore et al., 2015; Saraf‐Sinik et al., 2015; Sherman et al., 2017; Towal & Hartmann, 2006; Voigts et al., 2008), awake head‐fixed animals (Bermejo et al., 2002; de Kock & Sakmann, 2009; Deutsch et al., 2012; Guo et al., 2014; Hentschke et al., 2006; Hires et al., 2015; Isett & Feldman, 2020; O'Connor et al., 2010; Schwarz et al., 2010; Sumser et al., 2025; Urbain et al., 2015), and anesthetized preparations using motor nerve stimulations (Arabzadeh et al., 2005; Bagdasarian et al., 2013; Brown & Waite, 1974; Castro‐Alamancos & Bezdudnaya, 2015; Derdikman et al., 2006; Lottem & Azouz, 2008; Szwed et al., 2003; Yu et al., 2015; Zucker & Welker, 1969) and optogenetic methods (Park et al., 2016). Passive whisker stimulations, in contrast, have been studied under anesthesia using mechanical manipulators (Furuta et al., 2020; Gibson & Welker, 1983; Haidarliu et al., 1999; Krupa et al., 2001; Kwegyir‐Afful et al., 2008; Kwon et al., 2018; Lichtenstein et al., 1990; Pinto et al., 2000; Shoykhet et al., 2000; Simons, 1978; Vilarchao et al., 2018; Welker et al., 1992) or air puff‐induced deflections (Ahissar et al., 2001; Ollerenshaw et al., 2012; Sosnik et al., 2001; Sumser et al., 2025).

In this study, active and passive deflections differ fundamentally in how they encode whisking phase. During active whisking, whisker protraction is accompanied by axial rotation (torsion) of the whisker together with the follicle, while the bending of the ES and whisker shaft's deflection remains in the azimuthal direction (see Figure 8). This leads to phase‐specific activation of radially organized mechanoreceptor subsets. In contrast, passive whisker deflections lack torsion, and any unidirectional whisker deflections consistently stimulate the same set of mechanoreceptors (Figure 10).

Neural data supports this distinction. In passive deflections studies, the trigeminal neurons (the cell bodies of the processing mechanoreceptors) responded only to changes in force direction, while during active whisking evoked by facial nerve stimulations, these neurons responded with a temporal pattern reflecting whisking phase (Szwed et al., 2003; Zucker & Welker, 1969). Similarly, in response to repetitive passive whisker stimulation in awake head‐fixed mice, thalamic neurons showed nearly identical raster plots, while the active whisker movements, including object contact, provoked a clear phase‐dependent activity (Sumser et al., 2025). In the cortex, directionally tuned responses to passive stimulation have been documented in layers 2/3 and IV (Andermann & Moore, 2006; Kremer et al., 2011; Kwon et al., 2018), and voltage‐sensitive dye imaging has revealed direction‐specific activation patterns in the rat barrel cortex (Tsytsarev et al., 2010).

These studies demonstrate that passive whisker movement in different directions activates mechanoreceptor subsets arranged radially around the whisker shaft, each corresponding to a specific direction. During active whisking, the internal shaft's bending varies with torsional motion, stimulating different mechanoreceptor sets and generating phase‐coded activity patterns across the somatosensory pathway.

We thus propose that the follicle's pre‐neuronal mechanical processing differentiates active from passive sensing. In active whisking, the torsion‐influenced bending of the ES engages sequentially different sets of mechanoreceptors that encode whisking phases. In passive sensing, the absence of torsion yields a fixed activation pattern. This distinction is reflected at every stage of the sensory pathway.

### 4.3 Mechanical forces and shaping of follicular morphology

Our structural findings suggest that repeated mechanical stress may shape the follicle during development. In adult rats and mice, the ES and proximal part of the follicle display rostral resting curvature that is most prominent in the caudal whiskers (Figures 5 and 6). This morphological gradient likely facilitates the greater movement amplitude reported of caudal whiskers (Berg & Kleinfeld, 2003; Carvell & Simons, 1990; Wineski, 1983), which in turn influences local mechanical loads.

We propose that torsional deformation of the ES and proximal part of the follicle, particularly in caudal whiskers, contributes to the increased whisking amplitude and greater curvature of these whiskers. This hypothesis aligns with the idea that anatomical gradient produces unique kinematic signatures across whiskers and thereby their functional specialization that facilitates edge detection and texture discrimination (Gugig et al., 2020; Hobbs et al., 2016).

Although follicular curvature has not been emphasized in prior morphological studies, it is visible in published figures across multiple datasets from both rats (e.g., Furuta et al., 2020, figures 1 and 3, and graphic summary; Kim et al., 2011, figure 2a; Luo et al., 2021, figure 4, left) and mice (Jhaveri et al., 1998, figures 4A and 8A; Oshima et al., 2001; Tang et al., 2019, figure 1B; Whiteley et al., 2015, figure 1H; Zhang et al., 2024, figure 4). This curvature of the proximal end is also visible in dissected whisker follicles (Ghoroghi et al., 2013, figure 1a) and in the images of isolated and sliced follicles (Lin et al., 2015, figure 1B1). These features likely went unremarked, but the patterns they suggest nonetheless demonstrate conserved morphological adaptations.

Interestingly, this curvature is absent in newborns and early postnatal stages (Davidson & Hardy, 1952; Kollar, 1966), when the whiskers are thin, keratinized, and not under voluntary motion control (Erzurumlu & Gaspar, 2012). Yet, even passive contacts occurring during early development (Sullivan et al., 2003) may generate sufficient internal forces to drive the morphological shaping of the follicle. Similar morphogenetic mechanisms were reported in other developing epithelial‐derived structures, where mechanical forces guide shape formation (Mammoto et al., 2013; Nelson, 2016).

Since the caudal whiskers are thicker and longer, they likely experience greater mechanical stress, reinforcing the observed curvature gradient. We therefore suggest that mechanical stress during early life shapes the curvature of both the follicle and the ES through developmental morphogenesis. This contributes to a structurally encoded gradient that supports functional tactile specialization.

## 5 CONCLUSION

We identified a flexible, keratin‐based ES within the basal part of the intermediate portion of the whisker shaft. This segment easily deforms, enabling torsion and deflection of the whisker shaft and leading to consecutive, phase‐dependent stimulation of mechanoreceptors within the follicle. This mechanistic insight bridges anatomical, mechanical, and functional levels of analysis and provides a coherent model for whisking phase encoding. The curvature and resilience of the ES and follicular structures likely emerge from developmental morphogenesis shaped by mechanical stress, ensuring both fidelity and adaptability of the sensory system.

## AUTHOR CONTRIBUTIONS

**Sebastian Haidarliu:** Conceptualization; writing – original draft; investigation; methodology; data curation. **Guy Nelinger:** Methodology; data curation; formal analysis; investigation. **Luka Gantar:** Investigation; methodology; formal analysis; data curation. **Ehud Ahissar:** Conceptualization; supervision; funding acquisition; writing – review and editing. **Inbar Saraf‐Sinik:** Conceptualization; writing – review and editing; methodology; funding acquisition; investigation; data curation; resources.

## FUNDING INFORMATION

The United States‐Israel Binational Science Foundation (BSF, grant no. 2021327); The European Research Council (ERC) under the EU Horizon 2020 Research and Innovation Programme (grant no. 786949); the Israel Science Foundation (ISF, grant no. 2237/20); The Weizmann‐UK Collaboration and a research grant from the Estate of Thomas Gruen.

## CONFLICT OF INTEREST STATEMENT

The authors declare no conflicts of interest.
